# Novel Magnetic Attachment System Manufactured Using High-Frequency Heat Treatment and Stamp Technique: Introduction and Basic Performance

**DOI:** 10.3390/dj10050075

**Published:** 2022-05-02

**Authors:** Adityakrisna Yoshi Putra Wigianto, Yuichi Ishida, Takashi Matsuda, Takaharu Goto, Megumi Watanabe, Tetsuo Ichikawa

**Affiliations:** Department of Prosthodontics & Oral Rehabilitation, Graduate School of Biomedical Sciences, Tokushima University, 3-18-15, Kuramoto, Tokushima 770-8504, Japan; c302051011@tokushima-u.ac.jp (A.Y.P.W.); matsuda.takashi.1@tokushima-u.ac.jp (T.M.); tak510@tokushima-u.ac.jp (T.G.); megwat@tokushima-u.ac.jp (M.W.); ichi@tokushima-u.ac.jp (T.I.)

**Keywords:** dental magnet, overdenture, magnetic retention, denture retention, denture attachment

## Abstract

Recently, a novel magnetic attachment with extremely low cost and high performance was developed. This article aims to introduce a novel magnetic attachment and to evaluate its basic eligibility for denture retention in clinical practice. The novel magnetic attachment system used in this study was the direct-bonding root-keeper-type Magteeth™ MT800 (MagneDesign, Nagoya, Japan). The retentive force without displacement (position 0) and after horizontal displacement to positions 0.5, 1, 1.5, 2, 2.5, and 3 mm were measured. The values relative to the retentive force without displacement were gradually decreased to 82.7 ± 16.3%, 68.8 ± 17.1%, 62.4 ± 15%, 47.2 ± 13.1%, 35.7 ± 9.9%, and 20.7 ± 6.5%, respectively. The retentive force and magnetic field strength did not change significantly after the load test (100 N load, 10,000 times). No new gap between the metal and resin was found in the root keeper- and magnet assembly-embedded blocks after the load test. Some scratches on magnetic assembly and root keeper surface, while no change in the resin texture after the load test were observed. Based on the findings of this preliminary study, this novel low-cost magnetic attachment exhibited favorable retention, strength, and durability for clinical use.

## 1. Introduction

Patients requiring prosthodontic treatment do not always present with ideal alveolar ridge conditions, often needing additional efforts than those needed for conventional removable denture fabrication by the dentist to achieve adequate retention and stability. In such cases, prosthodontic magnetic attachments are alternatives to conventional clasps in removable dentures for providing the required retention and stability [1,2,3,4].

Magnetic attachments provide several additional advantages over other attachment types. They require minimal occlusal space owing to their small size, can be easily detached by exerting excessive lateral forces, are forgiving in need of parallelism, can be easily cleaned, and are easy to deliver and use by dentists and patients, respectively [2,3,5]. Magnetic attachments provide retention by utilizing the attractive forces between two magnetic components: the magnetic assembly (MA) embedded in the denture base and the keeper fixed in the abutment tooth [4]. MA and keeper designs can be classified according to the magnetic field system (closed or open), magnetic arrangement (sandwich or cap), keeper form (metal cast, direct-bonding root keeper (RK), or screw for implant), and attachment surface design (flat, dome, cushion, or precision) [6,7].

The latest advance or modification in the dental magnetic attachment system was using Nd–Fe–B as a hard magnetic material and replacing epoxy seal with laser welding [4,8]. Over the past five years, magnetic attachment research has mostly focused on clinical outcomes [9], its use for implant attachment as compared to other attachments [10], or measurement standard settings evaluation [11,12,13]. There were no articles available about magnet assembly or structure innovation. Recently, a novel magnetic attachment system was developed, which is economical and has excellent performance. The purpose of this article is to introduce this novel magnetic attachment and to evaluate the basic eligibility of the novel magnetic attachment for denture retention in clinical practice.

## 2. Novel Magnet Assembly

**Figure 1:**
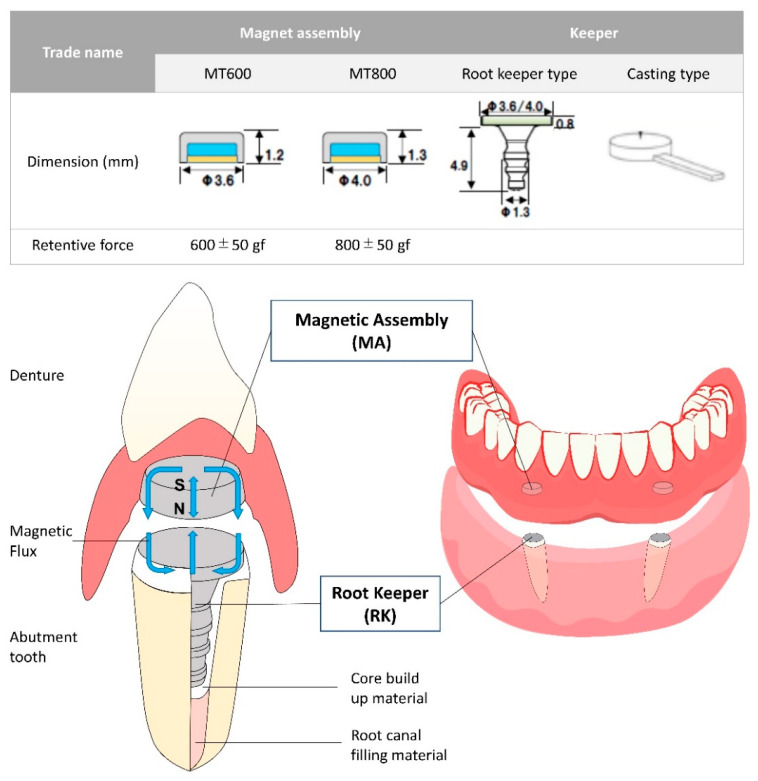
Illustration of the novel magnetic attachment system.

Figure 1 shows the novel magnetic attachment system (Magteeth™, Magnedesign, Nagoya, Japan), which has two types of MA and keeper (RK and casting types). The RK type can be easily incorporated in the denture since the keeper already consists of prefabricated intra-radicular post components [3,6]. This design enables direct bonding to the tooth without additional laboratory casting procedures, thereby eliminating the risk of magnetic keeper distortion during the casting process, which decreases the retentive force. Moreover, the easy and fast approach enables the conversion of an existing conventional denture to a magnet-retained one in a single visit [14].

**Figure 2:**
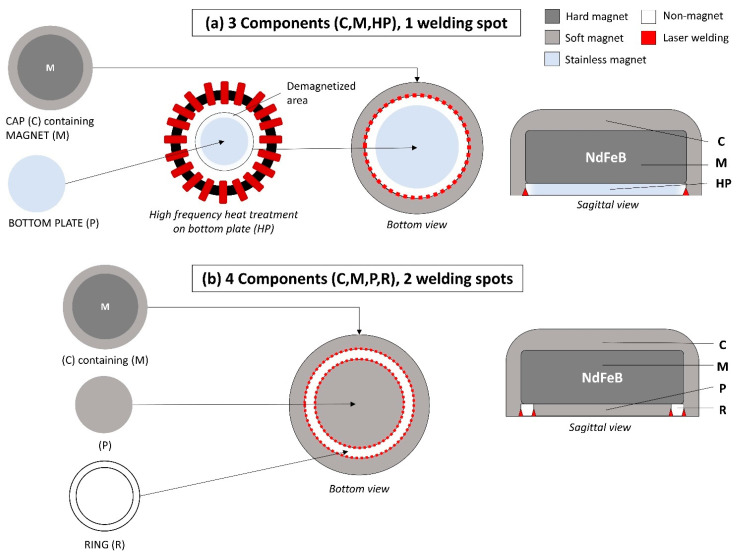
Structural differences between the novel magnetic attachment (**a**) and the conventional magnetic attachment (**b**).

Figure 2 demonstrates the technical differences between the conventional and novel MAs. The novel MA is manufactured as a cup-yoke type, with neodymium–ferrite–boron (Nd–Fe–B) as the magnetic component, surrounded by a cup of ferritic stainless steel (SUS434) and a bottom plate (SUS304), and sealed using the laser-welding technique (Figure 2a). The previous generation MA was manufactured by sealing the hard-magnetic material (Nd–Fe–B magnet) inside soft-magnetic components (cap and the bottom plate) and a non-magnetic ring (Figure 2b). Furthermore, the cap, bottom plate, and non-magnetic ring were attached and sealed by laser welding. A non-magnetic area is essential for magnetic circuit generation, enhancing the retentive force between the MA and keeper. By using the novel magnetic attachment, the non-magnetic area can be generated by high-frequency heat treatment to the bottom plate that demagnetizes the circumferential edge area, thus eliminating the need for a non-magnetic ring component. Therefore, this novel magnetic attachment simplifies the MA from four components with two welded joints to three components with one welded joint. In addition, the retentive force improves owing to the magnetization of the bottom plate. Moreover, each component of the previous generation magnetic attachment was manufactured by milling, while all components of the novel magnetic attachment were manufactured using stamping; thus, the production cost can be reduced significantly.

## 3. Materials and Methods

The novel magnetic attachment system used in this study was the direct-bonding RK-type Magteeth™ MT800 system.

All surfaces, except flat MA-RK surfaces, were sandblasted using 50 µm alumina powder at 0.3 MPa pressure for 3 s, followed by steam cleaning. Metal primer (Metal Primer Z, GC, Tokyo, Japan) was applied thoroughly to the sandblasted surfaces. Rectangular resin blocks (30 × 25 × 8 mm) were prepared to affix the MA and RK in a universal testing machine (UTM: AG-1KNX Precision UTM, Shimadzu, Kyoto, Japan). The RK was cemented using a dual-polymerizing composite cement (Clearfill^®^ DC Core Automix^®^ ONE, Kuraray Noritake Dental Inc., Tokyo, Japan), while the MA was fixed in the resin blocks using auto polymerizing acrylic resin (PROVINICE, Shofu, Kyoto, Japan) such that the surface of the MA/RK was flushed with that of the resin block. Five block specimens with each MA/RK were prepared.

### 3.1. Retentive Force at Horizontally Displaced Positions

**Figure 3:**
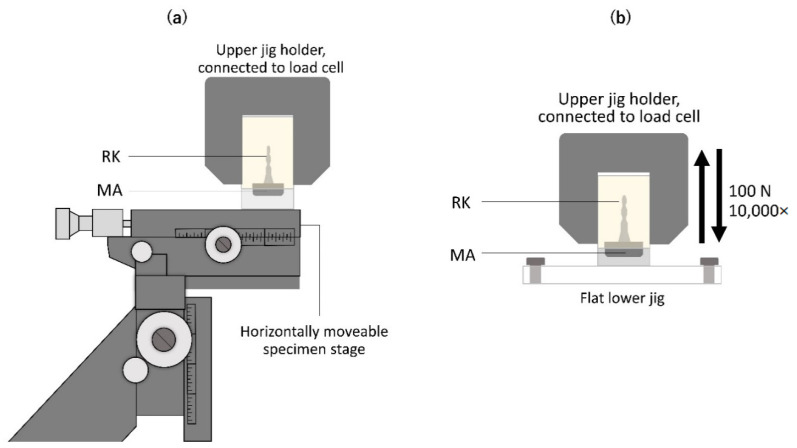
Experimental settings on the universal testing machine to evaluate the retentive force at displaced positions (**a**) and to perform the load test (**b**).

The RK block specimen was fixed to the upper jig of the UTM, whereas the MA block was fixed to the lower part with an adjustable X-, Y-, and Z-axes stage (No. 7674, Narishige, Tokyo, Japan; accuracy 100 µm) using a cyanoacrylate adhesive (Aron Alpha™, Toagosei, Tokyo, Japan; Figure 3a). The initial retentive force was measured at a horizontal displacement of 0 mm (position 0). The retentive force was measured by evaluating the greatest force needed to separate the RK from the MA at a crosshead speed of 5 mm/min; this was repeated five times for each position. Furthermore, the stage with the MA block was horizontally displaced to evaluate the retentive forces at 0.5, 1, 1.5, 2, 2.5, and 3 mm (positions 0.5, 1, 1.5, 2, 2.5, and 3, respectively).

### 3.2. Durability Test

The MA block was fixed to a flat acrylic stage at the base of the UTM using a cyanoacrylate adhesive. The mode of the UTM was set to cyclic load, applying 100 N force 10,000 times with a crosshead speed of 15 mm/min (Figure 3b). The retentive force and magnetic field strength were measured before and after the load test. The greatest magnetic field strength around the MA was measured using a magnetometer (MG-501PRB probe attached to an MG-501 Gaussmeter, Magna, Tokyo, Japan). Five measurements were performed for each specimen before and after the load test.

The surface conditions of the MA/RK resin blocks before and after the load test were morphologically examined using a digital stereomicroscope (VHX-F, Keyence, Osaka, Japan) at 50× and 150× magnifications.

### 3.3. Statistical Analysis

Statistical analysis was carried out using the SPSS v25 statistical software (IBM, New York, USA). The retentive forces at the displaced positions were compared using one-way analysis of variance (ANOVA) and Tukey’s multiple comparisons at 95% confidence interval (α = 0.05). The paired t-test was performed to analyze the difference in retentive force and magnetic field strength before and after the load test at 95% confidence interval (α = 0.05).

## 4. Results

**Figure 4:**
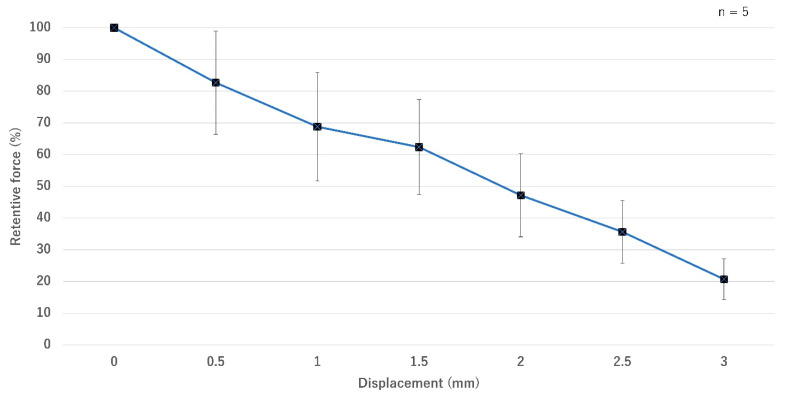
Comparisons of retentive forces at displaced positions (means and standard deviations).

The retentive force without displacement (position 0) and before the load test was defined as 100%. After horizontal displacement to positions 0.5, 1, 1.5, 2, 2.5, and 3, the relative values were gradually decreased to 82.7 ± 16.3%, 68.8 ± 17.1%, 62.4 ± 15%, 47.2 ± 13.1%, 35.7 ± 9.9%, and 20.7 ± 6.5%, respectively (Figure 4). The result of one-way ANOVA showed that the retentive force decreased significantly from position 1 onward (*p <* 0.05).

**Figure 5:**
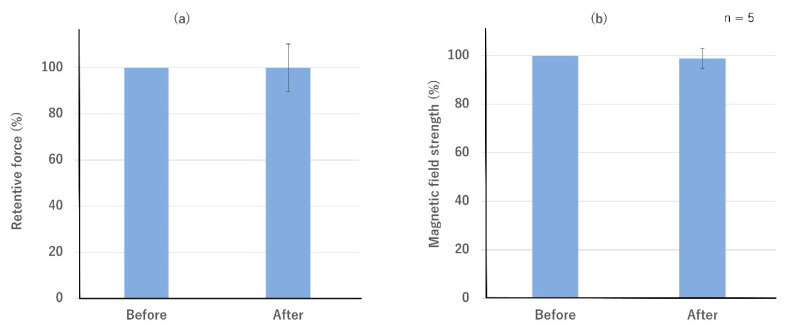
Comparison of (**a**) retentive force and (**b**) magnetic field strength after load test (means and standard deviations). No change was observed.

The relative value of retentive force after the load test was 100 ± 10.3% (Figure 5a). The relative value of magnetic field strength after the load test was 98.9 ± 4.1% (Figure 5b). No significant differences were found between the before- and after-values. The relative value of magnetic field strength 40 days later was 100.2 ± 2.5% and remained unchanged.

**Figure 6:**
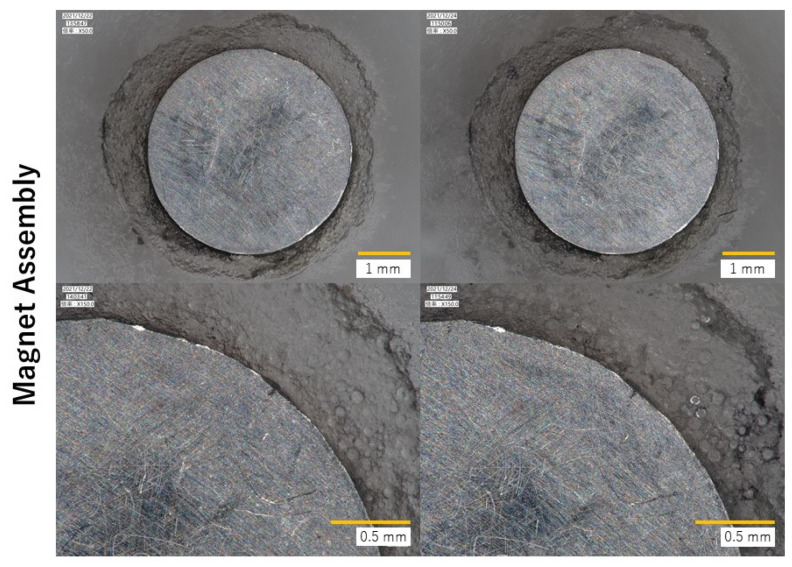

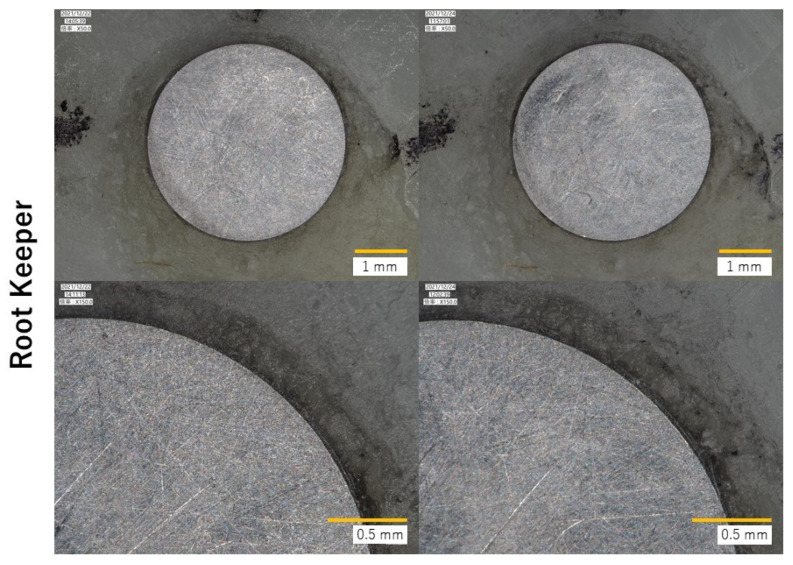
Surface evaluation of MA and RK between before (**left**) and after (**right**) load test. Upper: 50×, lower: 150× magnification.

Figure 6 shows the typical surface images of the MA/RK resin blocks before and after the load test. No new gaps were developed between the metal and the resin in the MA/RK blocks after the load test. Some scratches were observed on the surfaces of both MA and RK after the load test; however, no change in the resin texture was observed.

## 5. Discussion

Since first introduced, magnetic attachments used in prosthetic dentistry have developed considerably in terms of the magnetic material, housing, design of the soft-magnetic material, and sealing technique. The novel magnetic attachment used in this study was manufactured using three new approaches: demagnetization of the circumferential edge of the bottom plate using high-frequency heat treatment, stamping manufacturing of each component, and magnetizing the bottom plate. Because heat treatment was applied to demagnetize the edge of the bottom plate in this MA, the additional need for a non-magnetic ring component was eliminated. Thus, the welding spot was reduced to a one-line spot (cap-plate) compared to the two-line spots in the conventional MA (cap-ring and ring-plate). Laser welding was used to seal the hard magnet in the soft-magnetic yoke against oral fluids [4,15]. The reduction in laser-welding spots lowers the risk of seal breakage. Moreover, stamping manufacturing, as used in this novel procedure, reduces production costs. MA performance is related to size, the strength of magnetic force, little influence of inclinations and X/Y misalignment, and durability. The above novel manufacturing is expected to improve overall performance compared to those currently available magnetic attachment [16,17,18,19]. We performed in vitro experiments to confirm whether this novel magnetic attachment has adequate retentive force and durability for clinical use.

The retentive force of the magnetic attachment varies according to assembly size and design and the nature of hard magnetic material. Previous studies [11,12,13,16,17] showed that the retentive force of commercially available magnetic attachment with various diameters, thicknesses, and designs ranged from 1.37–6.57 N. The novel magnetic attachment tested in this study provides an equivalent or more retentive force than previous generations of attachments, at an MA/RK vertical height of approximately 2 mm.

Additionally, the actual measured retentive force was lower than the value described by the manufacturer as those reported in previous studies [16,17]. The retentive force depends strongly on the positional relationship between the MA and RK and the crosshead speed of the UTM, and achieving the ideal position according to the manufacturer’s instructions is difficult. The present study used the relative values before and after the intervention.

A special feature of magnetic attachments is auto-reseating of dentures using magnetic attraction. However, such attachments are sensitive to even a slight gap or displacement, leading to a reduction in retentive force [18,19]. Despite a significant reduction in retentive force at position 1 or displaced by 25% of the diameter, the retentive force was still 68.7%. According to Tanaka et al., the retentive force of the same cap-type MA was reduced by approximately 45% after a horizontal displacement of 25% [19]. Our novel magnetic attachment works with equal or better efficiency than conventional magnetic attachments during slight denture movement caused by chewing. Another measure to evaluate the magnetic attachment’s auto-reseating ability is assessing its retentive force in several inclined positions [20]. However, the measurement procedure is very complicated and difficult. We only evaluate the displacement on X/Y axis since there are more data for various magnetic attachment systems [19].

Several prosthodontic complications may be evident in magnetic attachment treatment, such as reduction in the retentive force of the magnetic attachment and desorption of magnetic attachments from the denture or abutment teeth. The retentive force may be reduced by the change in the MA/RK positional relationship leading to magnet corrosion following seal breakage due to scratches and deformation of the MA. The desorption of magnetic attachments from dentures and abutment teeth is caused by the deterioration of the cement around the magnetic attachment and the deformation of the denture base. The MA and keeper are repeatedly subjected to mechanical stresses by occlusal forces. Such repetitive mechanical stresses may lead to deterioration of the cement around the magnetic attachment and seal breakage. The retentive force of the magnetic attachment may be reduced significantly after several cycles (5000–20,000 times) owing to gap formation or surface deformation [21]. Therefore, the load test in this study (a load of 100 N applied 10,000 times) was performed to evaluate the durability of the magnetic attachment using the retentive force, magnetic field strength, and microscopic observation of the surface conditions of the magnetic attachment and its surroundings. The magnetic retentive force is based on magnetic field strength [6]. The maximum magnetic field strength on the surface of the MA was measured to evaluate the influence of the load test on the MA. Contrary to the results reported by Hao et al. [21], there were no significant changes in the retentive force and magnetic field strength before and after the load test. Although the load of 100 N applied 10,000 times in this study may be limited for testing the durability, this novel RK magnetic attachment was found to have sufficient durability to be used in patients.

Nevertheless, other aspects in this preliminary in vitro study that were not evaluated, such as corrosion resistance, durability in a greater number of load cycles, thermal cycles, and performance evaluations after incorporation in a denture, should be considered. Besides that, the durability test in this study was conducted in dry conditions. Therefore, evaluations in a wet condition or clinical test of this novel magnetic attachment system are required as the next step.

## 6. Conclusions

Based on the findings of this preliminary study, this novel low-cost magnetic attachment manufactured using a novel technology exhibited favorable retention strength and durability for clinical use.

## Data Availability

Not applicable.
